# Src tyrosine kinase promotes cardiac remodeling induced by chronic sympathetic activation

**DOI:** 10.1042/BSR20231097

**Published:** 2023-10-27

**Authors:** Wenqi Li, Yuzhong Zhu, Wenjing Wang, Dan He, Lei Feng, Zijian Li

**Affiliations:** 1 Wuxi School of Medicine, Jiangnan University, Wuxi, China; 2 Department of Cardiology and Institute of Vascular Medicine, Peking University Third Hospital; Beijing Key Laboratory of Cardiovascular Receptors Research; State Key Laboratory of Vascular Homeostasis and Remodeling, Peking University; NHC Key Laboratory of Cardiovascular Molecular Biology and Regulatory Peptides, Peking University; Research Unit of Medical Science Research Management/Basic and Clinical Research of Metabolic Cardiovascular Diseases, Chinese Academy of Medical Sciences. Beijing 100191, China; 3 Department of Pharmacy, Peking University Third Hospital, Beijing, China

**Keywords:** β-adrenergic receptor, cardiac remodeling, cardiomyocyte, Src

## Abstract

Cardiac remodeling serves as the underlying pathological basis for numerous cardiovascular diseases and represents a pivotal stage for intervention. The excessive activation of β-adrenergic receptors (β-ARs) assumes a crucial role in cardiac remodeling. Nonetheless, the underlying molecular mechanisms governing β-AR-induced cardiac remodeling remain largely unresolved. In the present study, we identified Src tyrosine kinase as a key player in the cardiac remodeling triggered by excessive β-AR activation. Our findings demonstrated that Src mediates isoproterenol (ISO)-induced cardiac hypertrophy, fibrosis, and inflammation *in vivo*. Furthermore, Src facilitates β-AR-mediated proliferation and transdifferentiation of cardiac fibroblasts, and hypertrophy and cardiomyocytes *in vitro*. Subsequent investigations have substantiated that Src mediates β-AR induced the extracellular signal-regulated protein kinase (ERK1/2) signaling pathway activated by β-AR. Our research presents compelling evidence that Src promotes β-AR-induced cardiac remodeling in both *in vivo* and *in vitro* settings. It establishes the promoting effect of the β-AR/Src/ERK signaling pathway on overall cardiac remodeling in cardiac fibroblasts and underscores the potential of Src as a therapeutic target for cardiac remodeling.

## Introduction

Heart failure stands as a prominent contributor to global morbidity and mortality rates. It is widely recognized that cardiac remodeling serves as the underlying pathological foundation for various cardiovascular diseases. It is often regarded as a determinant of the clinical outcome in heart failure, significantly influencing disease progression and prognostic implications [1]. Cardiac remodeling is characterized by the development of cardiac hypertrophy and fibrosis. The clinical manifestations of heart remodeling include alterations in cardiac size, shape, and function [2]. Implementing effective interventions to target cardiac remodeling can enhance cardiac function and mitigate the risk of heart failure. Therefore, the identification of novel targets for the prevention and treatment of cardiac remodeling is of paramount importance.

Prolonged activation of the sympathetic system and increased plasma catecholamine levels have been implicated in the induction of pathological cardiac remodeling over the long term [3]. β-Adrenergic receptors (β-ARs), which belong to the G protein-coupled receptor (GPCR) family, represent the predominant receptors in the heart and are known to respond to elevated levels of plasma catecholamines [4]. The activation of β-AR signaling pathways plays a critical role in the progression of cardiac remodeling. Currently, β-blockers are widely utilized in clinical practice for the treatment of heart failure with reduced ejection fraction. These medications effectively counteract the excessive activation of the sympathetic nervous system on the heart, thereby ameliorating ventricular remodeling and enhancing cardiac function. They have emerged as fundamental therapeutic agents for cardiovascular diseases [5]. However, the mechanisms underlying β-AR-induced cardiac remodeling are intricate, and many aspects of these mechanisms still elude our understanding.

Src kinase is indeed an important tyrosine kinase involved in the downstream signaling of β-ARs, particularly in the context of the beta-arrestin pathway [6]. Traditionally, G protein signaling pathways were considered the primary mediators of GPCR signals [7]. However, recent research has revealed that β-arrestin can independently mediate multiple signaling events initiated by GPCRs [8]. Upon activation, GPCRs can initiate signaling through either the G protein pathway or the β-arrestin pathway. In the G-protein-independent pathway of β-AR, Src is recruited by β-arrestin, leading to the activation of various downstream signaling molecules. This includes important signal transduction molecules such as c-Jun, ERK1/2, phosphodiesterase, and diacylglycerol. Through this pathway, a wide range of physiological and pathological signals can be transmitted [9]. Furthermore, Src kinase is also involved in the transactivation of β-ARs, leading to the activation of receptor tyrosine kinases (RTKs) such as the epidermal growth factor receptor (EGFR) [10]. This process plays a significant role, and Src acts as a key mediator in this transactivation process [11]. Despite Src’s important role in β-AR downstream signaling pathways, its specific involvement in β-AR-mediated cardiac remodeling, particularly *in vivo*, remains unclear.

Our research findings indicate that Src kinase plays a critical role in ISO-induced cardiac remodeling. We observed that inhibition of Src using a specific inhibitor called 4-amino-5-(methylphenyl)-7-(t-butyl) pyrazolo-(3,4-d) pyrimidine (PP1) can effectively prevent cardiac remodeling *in vivo*. Further investigations revealed that Src inhibition can suppress β-AR-mediated proliferation and transdifferentiation of cardiac fibroblasts, and cardiomyocyte hypertrophy* in vitro*. Additionally, our results demonstrated that Src can be phosphorylated by the β-AR agonist ISO and subsequently mediate downstream signaling through extracellular signal-regulated protein kinase (ERK1/2) at the cellular level. These findings shed light on some of the mechanisms involved in ISO-mediated cardiac remodeling. Our study aims to explore the mechanisms of cardiac remodeling induced by chronic sympathetic activation. It provides additional evidence at the whole-animal level for Src's role in mediating chronic sympathetic nervous system-induced cardiac remodeling. We clarified Src’s response to ISO stimulation in two critical cell types, cardiac fibroblasts, and cardiomyocytes. Src can independently promote cardiomyocyte hypertrophy or cardiac fibroblast transdifferentiation, providing a theoretical basis for precise therapies at the cellular or genetic level. Src, as a key downstream molecule of β-AR, may become a potential target for the development of a new generation of β-AR blockers. With the in-depth progress of GPCR research, the diversity of GPCR activation has become a hot topic of interest. Src may play a critical role as a key node in GPCR's multiple activations, such as GPCR-related translocation activation and biased signaling.

## Materials and methods

### Animal experiments

All animal studies were approved by the Biomedical Research Ethics Committee of Peking University and were compliant with the National Institutes of Health Guide for the Care and Use of Laboratory Animals.

All animal experiments were conducted at the Institute of Vascular Medicine, Peking University Third Hospital. Animals were anesthetized with Lidocaine hydrochloride (Sangon Biotech, China) and killed by CO2 asphyxiation.

The male C57BL/6 mice (12 weeks) were obtained from Weitonglihua Company (Beijing, China). Mice were maintained on a 12h light/dark cycle in a room with controlled temperature (25°C±2°C), which had free access to food and water. Cardiac remodeling model was established by daily subcutaneous injection of 10 mg/kg/d ISO (Sigma-Aldrich, U.S.A.) for 2 weeks, while the control group was given equal volume of vehicle. During 2 weeks, 1.5 mg/kg/d PP1 (Sigma-Aldrich, U.S.A.), a specific inhibitor of Src tyrosine kinase, or dimethyl sulfoxide was intraperitoneally injected 3 times per week (The mortality rate, Supplementary Material S1). All animal experiments were approved by the Institutional Animal Care and Use Committee of Peking University Health Science Center.

### Echocardiography

Echocardiography analysis was performed 1 day after the last injection of ISO. Mice were anesthetized with isoflurane (Baxter International Inc., U.S.A.). Images were obtained by the Visualsonics high-resolution Vevo 2100 system (VisualSonics, Canada). The diastolic left ventricular posterior wall thickness (LVPW;d) and systolic left ventricular posterior wall thickness (LVPW;s) were measured to calculate the ejection fraction (EF) and fractional shortening (FS). Cardiac systolic function was represented by the values of EF and FS. All measurements were averaged from three consecutive cardiac cycles.

### Quantitative histological analysis

Mice were anesthetized and sacrificed after echocardiography analysis. The hearts were excised and weighed immediately after being washed with cold PBS. The cardiac tissues for histological and immunohistochemistry analysis were fixed with 4% paraformaldehyde for 12 h, dehydrated in 20% sucrose for 24 h, and then embedded in paraffin. Paraffin embedded hearts were cut into 5-µm-thick sections on a microtome and transferred onto glass slides. Then serial sections (5 μm thick) were stained with hematoxylin and eosin stain (H&E) for morphological analysis and picrosirius red (PSR) was used for the detection of fibrosis according to the manufacturer’s instructions. Myocyte cross-sectional area and the percentage of collagen area were measured by a quantitative digital image analysis system (Image-Pro Plus 8.0) using the photographs to evaluate cardiac hypertrophy and cardiac fibrosis.

### Transcriptional analysis

Total RNA was isolated from heart tissues using Trizol Reagent (Thermo Fisher Scientific, U.S.A.), and then 1 µg RNA was reverse transcribed into cDNA using the ImProm-II reverse transcription system (Promega, WI, U.S.A.) with random primers. Relative quantitation was performed using real-time PCR Master Mix kit-SYBR Green (TransGen Biotech, China). Relative mRNA levels were normalized to GAPDH, and then quantified using the comparative ∆CT method. The sequences of the primers used for RT-PCR are as follows:

**Table:** 

ANP (mouse)	F: 5′-CTTCCAGGCCATATTGGAG-3′,
	R: 5′- GGGGGCATGACCTCATCTT-3′;
BNP (mouse)	F: 5′-ACAAGATAGACCGGATCGGA-3′,
	R: 5′-AGCCAGGAGGTCTTCCTACA-3′;
Collagen-I (mouse)	F: 5′-GTAACTTCGTGCCTAGCAACA-3′,
	R:5′-CCTTTGTCAGAATACTGAGCAGC-3′;
Collagen-III (mouse)	F: 5′-TGGTCCTCAGGGTGTAAAGG-3′,
	R: 5′-GTCCAGCATCACCTTTTGGT-3′;
IL-1β (mouse)	F: 5′-TGCCACCTTTTGACAGTGATG-3′,
	R: 5′-AAGGTCCACGGGAAAGACAC-3′;
IL-6 (mouse)	F: 5′-CGGCCTTCCCTACTTCACAA-3′,
	R: 5′-TTCTGCAAGTGCATCATCGT-3′;
GAPDH (mouse)	F: 5′-ATGTTCCAGTATGACTCCACTCACG-3′,
	R: 5′-GAAGACACCAGTAGACTCCACGACA-3′;
ANP (rat)	F: 5′-CTGGACTGGGGAAGTCAACC-3′,
	R: 5′-CAATCCTACCCCCGAAGCAG-3′;
BNP (rat)	F: 5′-CGTTGCTGAGAGGCTAGTGC-3′,
	R: 5′-CAGCGGCGACAGATTAAGGA-3′;
GAPDH (rat)	F: 5′-TCTCTGCTCCTCCCTGTTCT-3′,
	R: 5′-TACGGCCAAATCCGTTCACA-3′.

The CT (threshold cycle) values obtained for genes of interest were normalized to concurrent measurement of GAPDH mRNA level, and fold changes were compared with the control.

### Cell culture

Neonatal rat cardiomyocytes (NRCMs), neonatal rat cardiac fibroblasts (NRCFs) and HEK-293A were cultured in DMEM medium, supplemented with 10% FBS and 1% penicillin and streptomycin, and maintained at 37°C with 5% CO_2_. 1- to 2-day old neonatal Sprague Dawley rats were purchased from Weitonglihua Company (Beijing, China). NRCMs and NRCFs were prepared as previously described [12].

### Plasmid transfection

When HEK-293A reached 60-80% confluence, related-gene transfer was started by plasmid transfection using Lipofectamine 2000 (Invitrogen, U.S.A.) as described by the manufacturer; After 36 h, total protein lysates were subjected to immunoblot analysis.

### Measurement of cell surface area

The cells were fixed with 4% para-formaldehyde for 15 min and washed thrice for 10 min with phosphate-buffered saline (PBS). The cells were then permeabilized with 0.5% Triton X-100 for 5 min and washed three times for 10 min with PBS. Then, the cells were stained with Alexa Fluor® 488 Phalloidin (no. 8878, Cell Signaling) for 45 min in the dark and washed three times for 10 min with PBS. After that, the cell nuclei were stained with the Hoechst reagent for 5 min in the dark and washed three times for 10 min with PBS. Finally, imaging was performed using a Zeiss LSM 880 microscope. Images were analyzed using the ImageJ software.

### Drug treatment in vitro

Before treatment with ISO or PP1, HEK-293A was starved for 12 h with serum-free medium, while NRCFs cells was starved for 24 h with serum-free medium. For PP1, cells were pre-treated for 1 h prior with PP1 to ISO treatment.

### Western blot analysis

Cells were lysed in protein lysis (1% deoxycholic acid, 10 mM Na4P_2_O_7_, 1% TritonX-100, 10% glycerol, 100 mM NaCl, 5 mM EDTA (pH 8.0), 20 mM Tris-HCl (pH 7.4), 0.1% SDS, 50 mM NaF, 1 mM Na_3_VO_4_, 1 mM PMSF, 10 mg/L aprotinin). Equal amounts of protein were separated on SDS-PAGE gels and transferred to nitrocellulose membranes. The following primary antibodies were used: anti-phospho-ERK1/2 (no. 4695, Cell Signaling), anti-ERK (no. 9102, Cell Signaling), anti-GAPDH (no. 2118, Cell Signaling), anti-phospho-Src^Y416^ (no. 2101, Cell Signaling), anti-Src (no. 2109, Cell Signaling), anti-flag (no. F9291, Sigma), anti-PCNA (no. sc-56, Santa), anti-α-SMA (no. ab5649, Abcam). After treated with corresponding horseradish peroxidase (HRP)-conjugated secondary antibodies (Zhongshanjinqiao, China), the protein bands were detected using an enhanced chemiluminescence System (Millipore, U.S.A.) and visualized on a GeneGnome chemiluminescent imaging system (Syngene, U.K.).

### Statistical analysis

Data were summarized as means ± standard error of mean (SEM). Differences between more than two groups were analyzed by one-way analysis of variance (ANOVA) or two-way ANOVA with Tukey’s post-hoc multiple comparison tests. *P-*values < 0.05 were considered to be of statistically significance.

## Results

### Inhibition of Src attenuates ISO-induced cardiac hypertrophy

**Figure 1:**
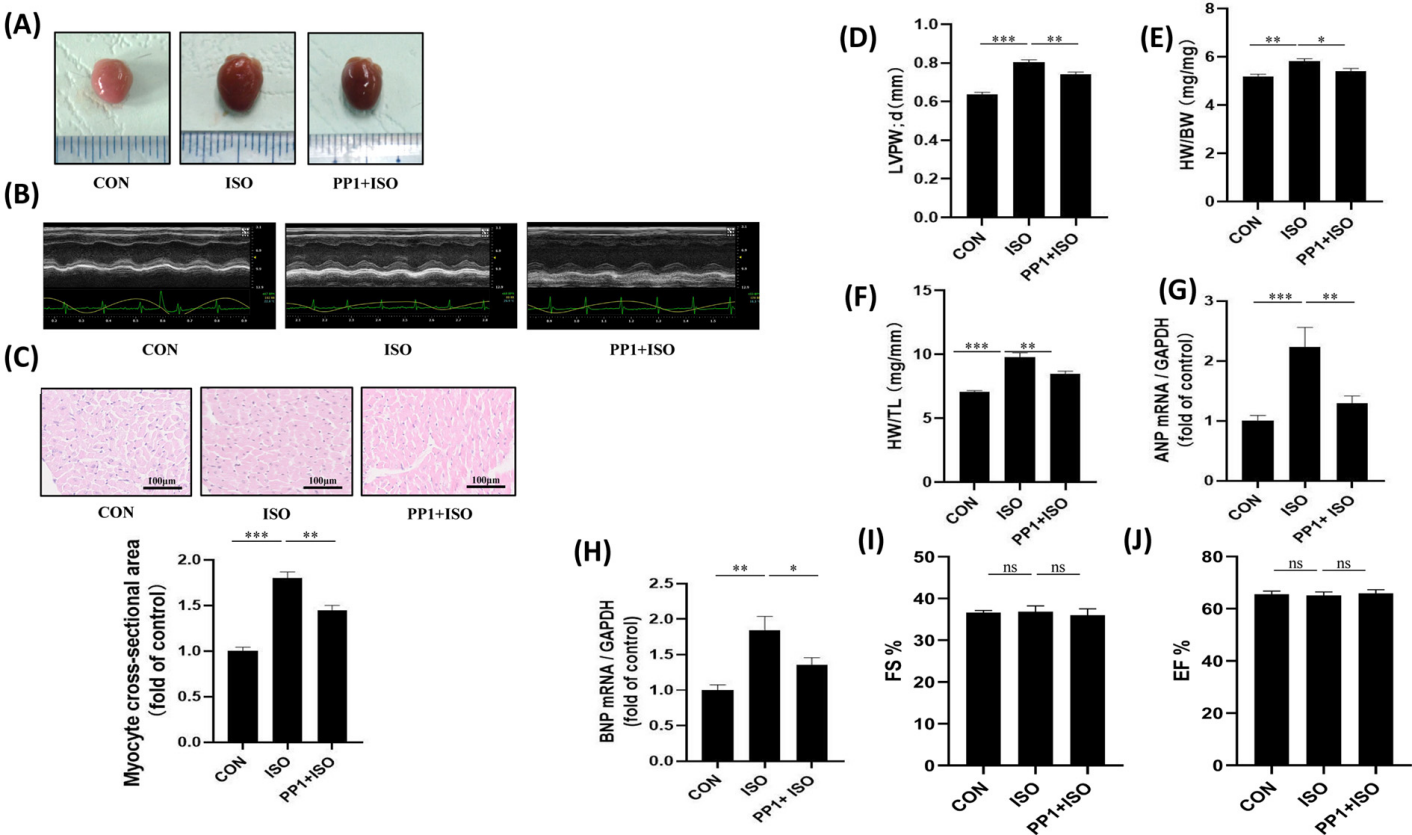
Inhibition of Src attenuates ISO-induced cardiac hypertrophy (**A**) Representative images of heart size. (**B**) Representative M-mode echocardiography images were taken to show left ventricular wall thickness. (**C**) Representative micrographs of myocyte cross-sectional area. Quantitative analysis of myocyte cross-sectional area; bar = 100 μm. *F* = 70.11, *P*<0.001. (**D**) Quantitative analysis of diastolic left ventricular posterior wall thickness; *F* = 50.20, *P*<0.001. (**E**) Quantitative analysis of HW/BW ratio; *F* = 32.23, *P*<0.001. (**F**) Quantitative analysis of HW/TL ratio; *F* = 65.61, *P*<0.001. (**G**) Quantitative analysis of ANP mRNA expression; *F* = 23.06, *P*<0.001. (**H**) Quantitative analysis of BNP mRNA expression; *F* = 27.61, *P*<0.001. (**I**) Left ventricular EF and (**J**) FS were measured to reflect cardiac contraction function; *F*(*I*) = 0.35, *P*(*I*)>0.05, *F*(*J*) = 0.17, *P*(*J*)>0.05. Data represent mean ± SEM. Analyzed by one-way analysis of variance (ANOVA) with Tukey’s post-hoc multiple comparison tests; *N*=7, *P<0.05, **P<0.01, ***P<0.001; ns, *P*>0.05.

Cardiac hypertrophy represents a significant pathological process in cardiac remodeling [13]. Several indicators are commonly used to assess cardiac hypertrophy, including the ratio of heart weight to tibia length (HW/TL), the ratio of heart-to-body weight (HW/BW), LVPW;d, myocyte cross-sectional area, as well as levels of atrial natriuretic peptide (ANP) and brain natriuretic peptide (BNP). These indicators provide valuable information for evaluating the presence and severity of cardiac hypertrophy and monitoring its progression over time [14]. To investigate whether Src mediates ISO-induced cardiac hypertrophy *in vivo*, we conducted a study where mice were pre-treated with the Src kinase inhibitor PP1. Our findings revealed that ISO administration led to significant alterations in various heart characteristics, including an increase in myocardial mass and ventricular wall thickness. Pre-treatment with PP1 resulted in a significant improvement in ISO-induced cardiac hypertrophy (Figure 1A,B,D). These results suggest that Src plays a crucial role in mediating ISO-induced cardiac hypertrophy and that its inhibition with PP1 can mitigate this effect. Furthermore, ISO exposure resulted in a significant increase in the ratios of HW/BW and HW/TL, a process reversed by PP1 treatment (Figure 1E,F). Similarly, ISO stimulation led to a significant up-regulation in the mRNA expression of ANP and BNP, which are markers of cardiac hypertrophy. PP1 treatment attenuated this increase in ANP and BNP expression (Figure 1G,H). Additionally, PP1 treatment reduced the myocyte cross-sectional area, which had been increased by ISO administration (Figure 1C). Assessment of cardiac function is crucial in the evaluation of cardiac diseases. The left ventricular EF and FS are considered key indicators for assessing cardiac contraction function. Our results indicated that the EF and FS were not significantly altered in the ISO-induced cardiac remodeling model, regardless of the administration of PP1 (Figure 1I,J). This means that the heart’s ability to contract is well preserved during the current cardiac remodeling phase.

### Inhibition of Src prevents ISO-induced cardiac fibrosis

**Figure 2:**
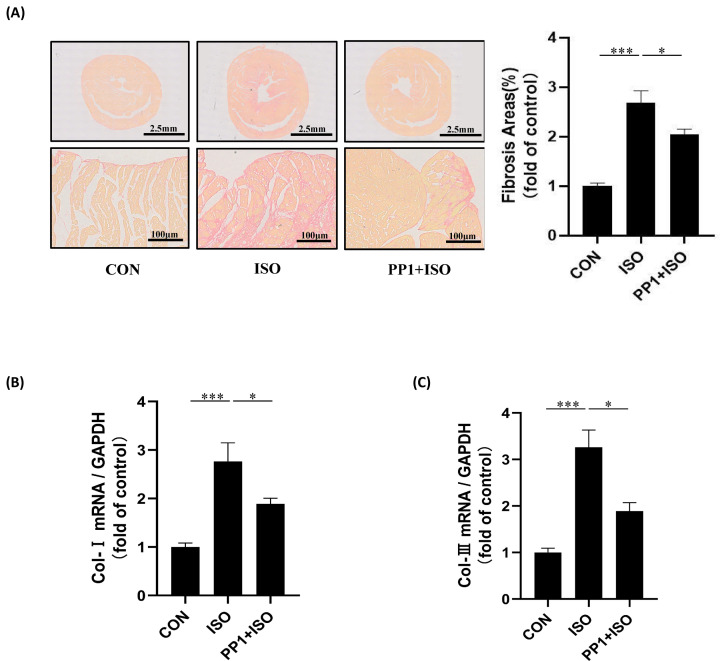
Inhibition of Src prevents ISO-induced cardiac fibrosis (**A**) Representative micrographs of picrosirius red-stained sections of the ventricle. Red parts represent collagen. The scale bars of 10× images are 2 mm, of 200× images are 100 μm. Quantification of cardiac interstitial collagen content from picrosirius red-stained sections with results expressed as the ratio of collagen area to heart area. There is no significance among all the groups; *F* = 35.76, *P*<0.001. (**B**) The mRNA expression of Collagen I in the heart tissue; *F* = 27.52, *P*<0.001. (**C**) The mRNA expression of Collagen III in the heart tissue; *F* = 42.56, *P*<0.001. Data represent mean ± SEM. Analyzed by one-way ANOVA with Tukey’s post-hoc multiple comparison tests; *N*=7, *P<0.05, ***P<0.001.

Chronic long-term ISO stimulation could cause fibrosis [15]. Our data showed that ISO led to cardiac fibrosis obviously and PP1 reduced this process (Figure 2A). Collagen I and Collagen III are also fibrosis markers. The mRNA expressions of Collagen I and Collagen III (Figure 2B,D) also revealed that ISO stimulation promoted cardiac fibrosis while inhibition of Src by PP1 ameliorated ISO-induced cardiac fibrosis. These results demonstrate that Src mediates β-AR-induced cardiac fibrosis.

### Inhibition of Src attenuates ISO-induced cardiac inflammation

**Figure 3:**
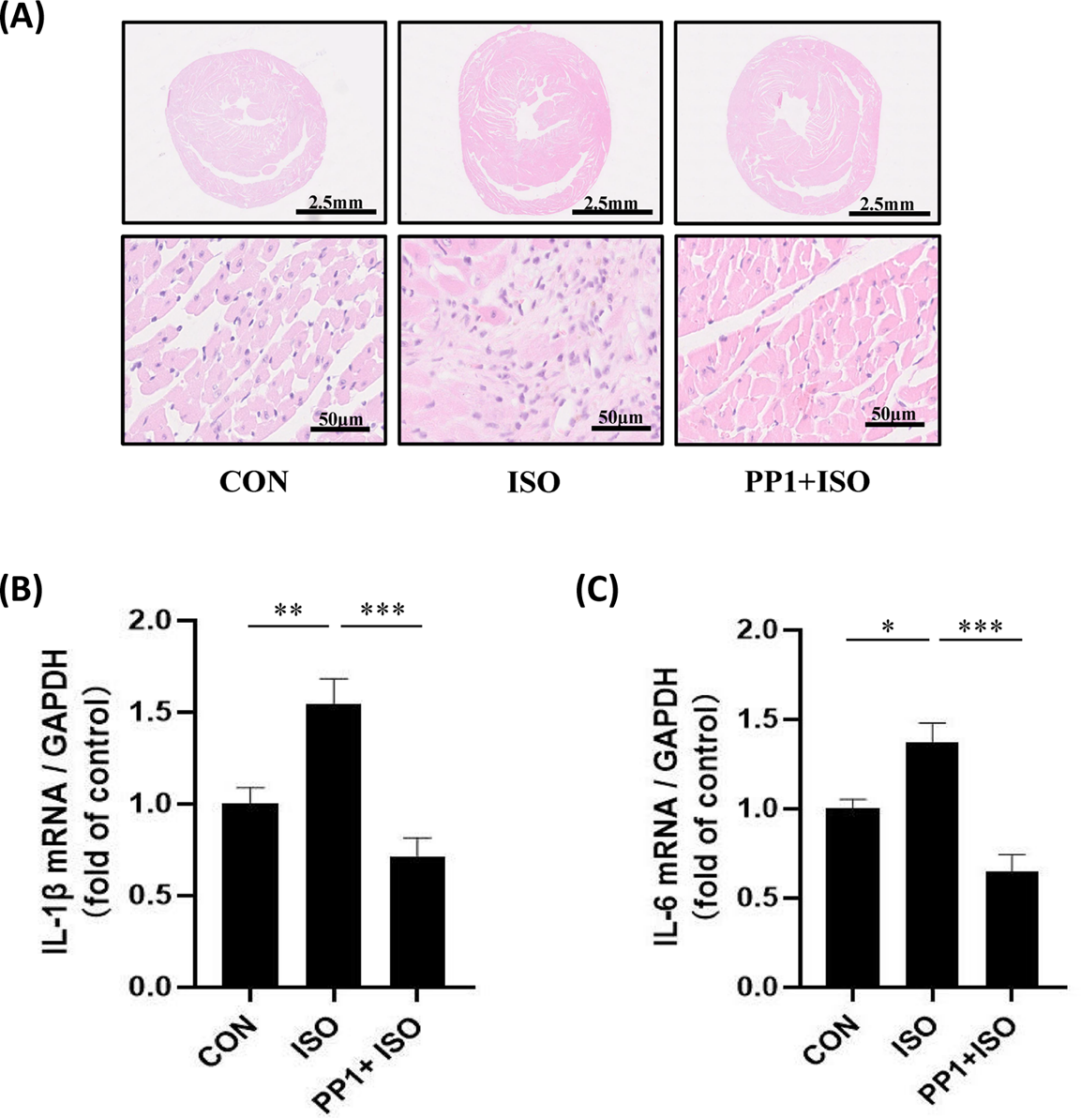
Inhibition of Src attenuates ISO-induced cardiac inflammation (**A**) Representative images showing H&E staining of heart sections. The arrows refer to the infiltrated inflammatory cells. The scale bars of 10× images are 2 mm, of 200× images are 100 μm. (**B**) The mRNA expression of IL-1β in the heart tissue; *F* = 36.03, *P*<0.001. (**C**) The mRNA expression of IL-6 in the heart tissue; *F* = 73.46, *P*<0.001. Data represent mean ± SEM. Analyzed by one-way ANOVA with Tukey’s post-hoc multiple comparison tests; *N*=7, *P<0.05, **P<0.01, ***P<0.001.

Cardiac inflammation is one of the main causes that results in cardiac fibrosis [16]. H&E staining showed that ISO-induced inflammatory cell infiltration. Our results indicated that PP1 can decrease chronic ISO-induced cardiac inflammation (Figure 3A). Furthermore, inflammatory cytokines and IL-6 were detected with real-time PCR. Consistent with cardiac fibrosis, PP1 significantly inhibited IL-1β and IL-6 production increased by ISO (Figure 3B,C). These results indicate that Src mediates β-AR-induced cardiac inflammation.

### Inhibition of Src reduces ISO-induced cardiomyocyte hypertrophy

**Figure 4:**
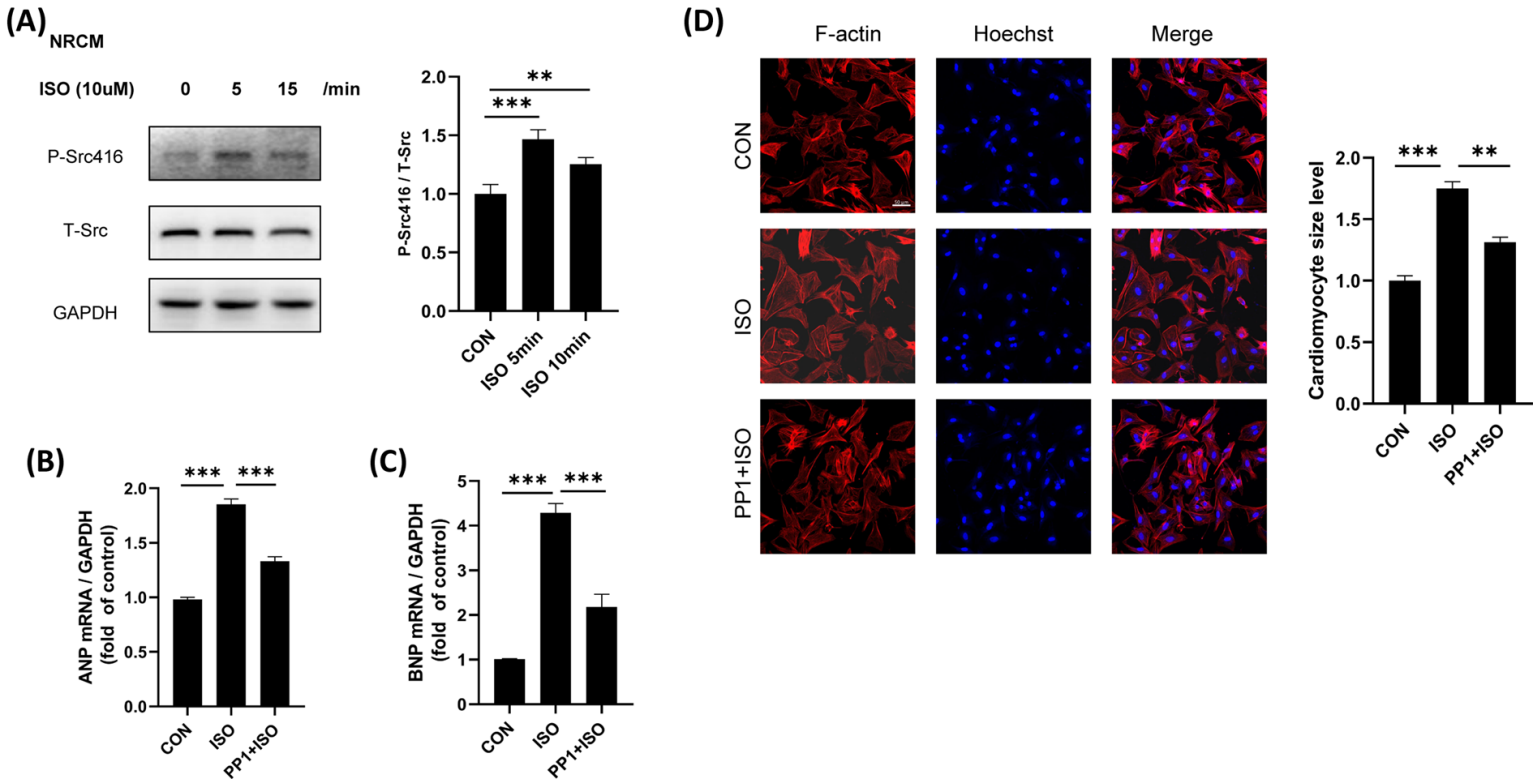
Inhibition of Src reduces ISO-induced cardiomyocyte hypertrophy (**A**) Src could be activated by isoproterenol. Cardiomyocytes were starved for 12 h, then stimulated with isoproterenol (10 μM) for 5 or 15 min, the phosphorylation of Src was analyzed using Western blotting. Activation of Src was determined by anti-Tyrosine 416 phospho-specific Src antibody. Experiments were performed in four times; *F* = 41.11, *P*<0.001. (**B**) The mRNA expression of ANP in the cardiomyocyte; *F* = 79.08, *P*<0.001. (**C**) The mRNA expression of BNP in the cardiomyocyte; *F* = 49.45, *P*<0.001. (**D**) Inhibition of Src reduces ISO-induced cardiomyocyte hypertrophy indicator F-actin. Cardiomyocytes were starved for 12 h, treated with or without PP1 (10 μM) for 1 h and then stimulated with isoproterenol (10 μM) in the presence or absence of PP1 for an additional 72 h; *F* = 67.44, *P*<0.001; bar = 50 μm. Data represent mean ± SEM. Analyzed by one-way ANOVA with Tukey’s post-hoc multiple comparison tests. *N*=4, number of NRCM>100 cells; *P<0.05, **P<0.01, ***P<0.001.

Cardiomyocyte hypertrophy is the main manifestation of cardiac remodeling. In vitro cardiomyocyte experiments, we found that ISO induced phosphorylation of Src Y416, the recognized Src kinase activation site. By analyzed the expression of myocardial hypertrophy marker ANP and BNP, we found that Src inhibitor PP1 can significantly reduce ISO-induced myocardial hypertrophy (Figure 4B,C). Similarly, cardiomyocyte size analysis showed that Src inhibitors significantly reduced ISO-induced increases in cardiomyocyte size (Figure 4D).

### Src inhibition prevents ISO-induced cardiac fibroblast proliferation and transdifferentiation

**Figure 5:**
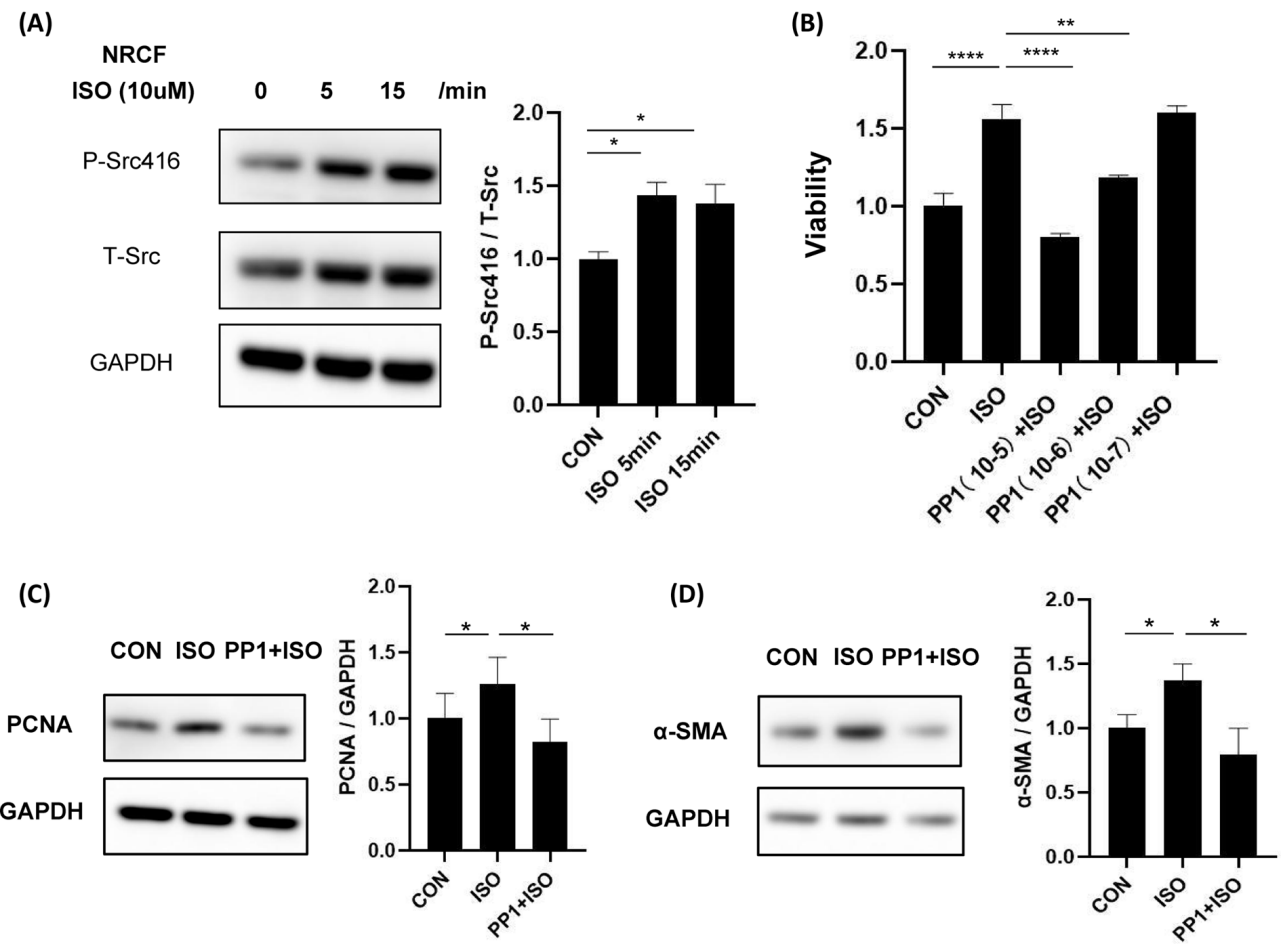
Src inhibition prevents ISO-induced cardiac fibroblast proliferation and transdifferentiation (**A**) Src could be activated by isoproterenol. Cardiac fibroblasts were starved for 12 h, then stimulated with isoproterenol (10 μM) for 5 or 15 min, the phosphorylation of Src was analyzed using Western blotting. Activation of Src was determined by anti-Tyrosine 416 phospho-specific Src antibody. Experiments were performed in four times; *F* = 5.12, *P*<0.05. (**B**) Inhibition of Src prevents ISO-induced cardiac fibroblasts cell viability. The cell viability was detected by CCK-8 assay; *F* = 10.58, *P*<0.001. (**C**) Inhibition of Src reduces ISO-induced cardiac fibroblasts proliferation indicator PCNA; *F* = 10.48, *P*<0.01. (**D**) Inhibition of Src reduces ISO-induced cardiac fibroblasts transdifferentiation indicator α-SMA; *F* = 30.81, *P*<0.001. Cardiac fibroblasts were starved for 12 h, treated with or without PP1 (10 μM) for 1 h and then stimulated with isoproterenol (10 μM) in the presence or absence of PP1 for an additional 24 h. Data represent mean ± SEM. Analyzed by one-way ANOVA with Tukey’s post-hoc multiple comparison tests; *N*=4, *P<0.05, **P<0.01, ***P<0.001.

Cardiac fibroblast proliferation contributes to cardiac fibrosis [17]. Cardiac fibroblast could response to different stimulators and regulates cardiac structure and function. Src could be activated by ISO in both 5 and 15 min (Figure 5A). Then we use CCK-8 to test NRCF proliferation. CCK-8 proliferation analysis revealed that ISO increased NRCF proliferation and PP1 inhibited ISO-induced NRCF proliferation in a dose-dependent manner (Figure 5B). ISO also promoted the expression of NRCF proliferation indicator, PCNA, and PP1 inhibited that (Figure 5C). We also revealed that PP1 inhibited ISO-induced transdifferentiation (Figure 5C). These data indicate that Src mediates β-AR-induced cardiac fibroblast proliferation.

### Src mediates β-AR-induced ERK1/2 signal pathway

**Figure 6:**
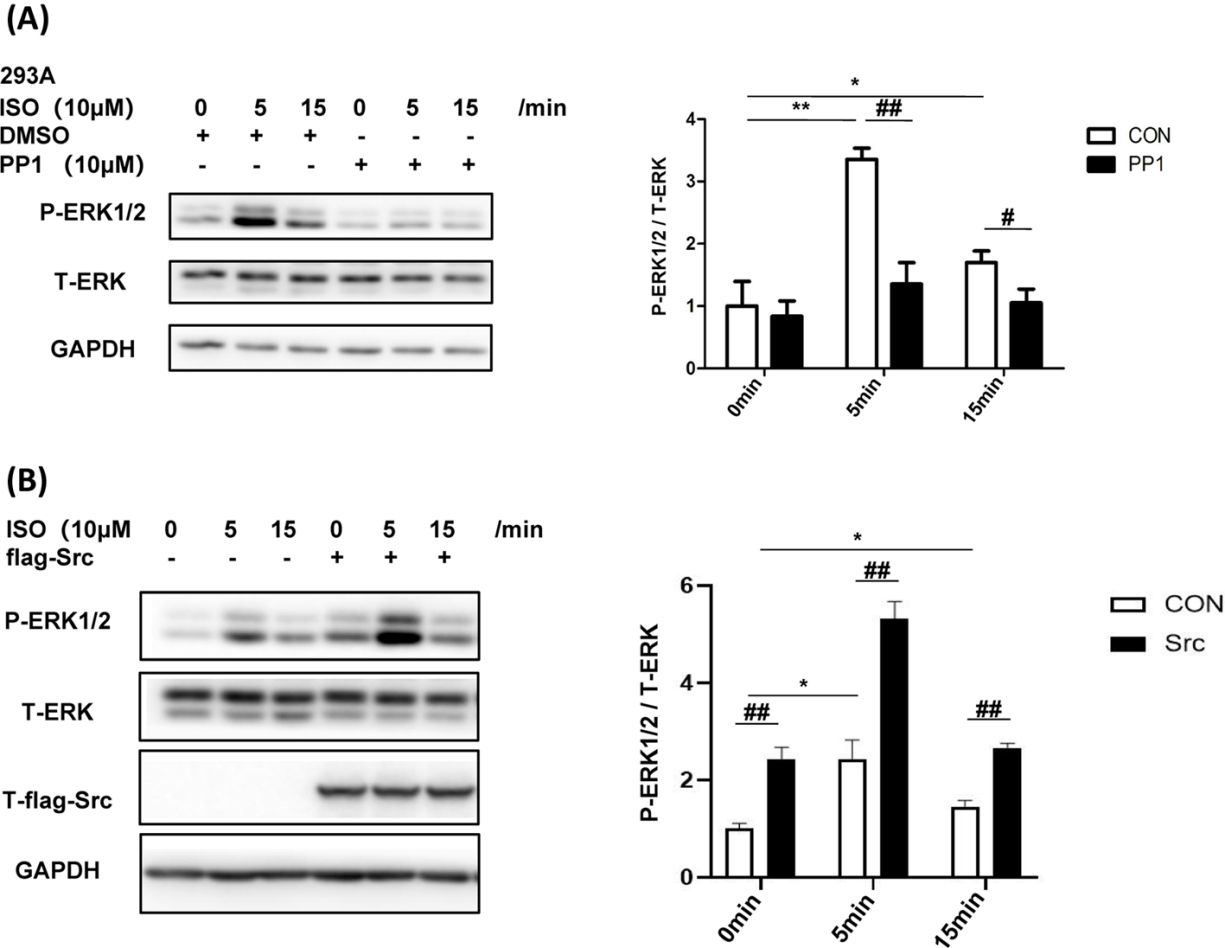
Src mediates β-AR-induced ERK1/2 signal pathway (**A**) Inhibition of Src decreases ISO-induced ERK1/2 phosphorylation. HEK-293A was starved for 12 h, treated with or without PP1 (10 μM) for 1 h, then stimulated with isoproterenol (10 μM) for 5 or 15 min; *F*(interaction) = 9.06, *P*<0.01. (**B**) Overexpression of Src increases ISO-induced ERK1/2 phosphorylation. HEK-293A was transfected with flag-Src plasmid, starved for 12 h, then stimulated with isoproterenol (10 μM) for 5 or 15 min; *F*(interaction) = 8.59, *P*<0.01. Data represent mean ± SEM. Analyzed by two-way ANOVA with Tukey’s post-hoc multiple comparison tests; *N*=4, *P<0.05, **P<0.01, #*P*<0.05, ##*P*<0.01.

It is well known that ERK1/2 pathway plays an important role in β-AR-mediated cardiac remodeling [18]. The results showed that inhibition of Src decreased the phosphorylation of ERK1/2 induced by ISO obviously (Figure 6A). Overexpression of Src increased ISO-induced ERK1/2 phosphorylation (Figure 6B). Taken together, β-AR/Src/ERK signal pathway plays an important role in cardiac remodeling.

## Discussion

Indeed, cardiac remodeling plays a critical role in the development and progression of numerous cardiovascular diseases [19]. It involves dynamic changes in the geometry, mass, volume, and function of the heart as an adaptive response to myocardial injury or alterations in hemodynamic load [20]. Long-term over-activation of the sympathetic nervous system and elevated plasma catecholamine levels have been identified as factors that can contribute to the pathological remodeling of the heart [21]. Therefore, understanding the underlying mechanisms involved in cardiac remodeling induced by excessive sympathetic system activation holds great significance for cardiovascular conditions characterized by cardiac remodeling. By unraveling these mechanisms, potential therapeutic targets and interventions can be identified to prevent or mitigate the adverse effects of pathological cardiac remodeling in various cardiovascular diseases.

β-ARs are prototypical members of the G protein-coupled receptor family, and they play a critical role in regulating various essential cellular processes [22]. The activation of β-AR signaling pathways is known to be significantly involved in the initiation and progression of cardiac remodeling [23]. In the cardiovascular system, the application of β-AR research primarily revolves around the use of β-blockers [24]. These medications function by competitively inhibiting β-ARs, leading to negative chronotropic, inotropic, and dromotropic effects. By reducing myocardial oxygen consumption and preventing excessive activation of sympathetic nerves in the heart, β-blockers have proven effective in improving ventricular and vascular remodeling and function [25]. The development of β-blockers has evolved from the first generation, represented by propranolol, to the current third generation, encompassing various selective and subtype-specific agents with improved efficacy and fewer side effects. Indeed, β-blockers generally play a beneficial role in cardiovascular treatment, but their targets and roles can vary [26].

The first generation of β-blockers, such as propranolol, are non-selective blockers with a high affinity for both β1-AR and β2-AR. However, the non-selective blockade of β2-AR receptors can lead to increased cardiac load and bronchospasm, limiting their usage [27]. With further research, second-generation β-blockers were developed, including selective β1-AR blockers like metoprolol. These agents improve target specificity, allowing for the avoidance of side effects on peripheral blood vessels and bronchial tubes. Carvedilol, a representative of third-generation β-blockers, has been widely used in clinical therapy. It is a non-selective blocker that targets both α1-AR and β-AR, including β1-AR and β2-AR [28]. By blocking α1-AR, third-generation β-blockers have vasodilatory effects, making them suitable for conditions like hypertension and congestive heart failure [29].

Despite the benefits of β-blockers usage in many patients, there are still challenges associated with their clinical application. Some patients may experience significant adverse reactions such as bronchospasm, peripheral vasospasm, and abnormalities in glucose and lipid metabolism [30,31]. These adverse effects are primarily due to insufficient receptor subtype selectivity and inadequate selectivity of downstream signaling pathways. As a result, further exploration of the pathogenesis of cardiac remodeling is necessary to identify new drug targets downstream of the receptors. Shifting the treatment approach from solely targeting the receptors themselves to targeting the downstream signaling pathways is crucial for precise receptor regulation.

Aiming at the above-mentioned problems in the application of drugs, we conducted mechanism studies and found that inhibition of Src can reduce ISO-induced cardiac hypertrophy, cardiac fibrosis, and cardiac inflammation. Src kinase is a key tyrosine kinase downstream of β-AR. Although Src plays such an important role downstream of β-AR at the signal level, its role in β-AR-mediated cardiac remodeling is unclear, especially at the animal level. We found that after a single subcutaneous injection of ISO (10 mg/kg) in mice for 14 consecutive days, the cardiac hypertrophy index, heart-body ratio, and heart-tibia ratio increased significantly, suggesting that the model was successfully established. After intervention with the Src inhibitor PP1 on the basis of ISO, we found that the hypertrophy index was significantly lower than that of ISO alone, indicating that Src can mediate ISO-induced cardiac hypertrophy.

However, existing studies have shown that Src kinase may be involved in the signal transduction of cardiomyocytes, cytoskeleton remodeling and stable regulation during cardiac hypertrophy [32]. Src can also activate endocytosis through the Shc-Grb2-Sos-Ras pathway by activating the βγ subunit of the G protein downstream of β1-AR, and activate ERK1/2, and leading to cardiac hypertrophy [33]. Similarly, Src kinase is a key factor in the anti-apoptotic effect regulated by β2-AR, acting through downstream signals of Gα1 and Gβγ, as well as upstream signals of PI3K [34]. In addition, the effects of β1-AR are often thought to be related to apoptosis [35]. Previous reports on β-AR have focused on *in vitro* studies. Studies have shown that Src mediates cardiac hypertrophy mainly through β1-AR [36], while β-AR isoforms in the heart are mainly β1-AR and β2-AR [37]. Whether cardiac hypertrophy has β-AR receptor subtype selectivity is worth exploring.

In addition, we found that the inflammation index in PPI-treated group was significantly lower than that of ISO alone, indicating that Src could mediate ISO-induced cardiac inflammation. At present, research on Src and cardiac inflammation is mainly focused on angiotensin II receptor type 1 (AT1R) [38]. Existing studies have shown that Src has been confirmed to be an upstream signal and a downstream signal of NADH oxidase by angiotensin II [39], and Src can enhance the up-regulation of oxidative stress-induced inflammatory factors [40]. Other experiments have pointed out that the activation of NADH oxidase is dependent on Src and plays an important role in the left ventricular hypertrophy and heart failure induced by angiotensin II [41]. In addition, the signaling pathway through which reactive oxygen species (ROS) leads to myocardial apoptosis, apart from the direct generation of tumor necrosis factor (TNF-α) by ROS, also involves the involvement of Src kinase [42]. Our research shows that Src mediates cardiac inflammation induced by β-adrenergic receptor over-activation.

MAPKs are a widely conserved family of serine/threonine protein kinases involved in multiple cellular processes, and their signaling pathways are fundamental for sustaining life [43]. They serve as key factors in signal transduction and regulation within cells. Among the MAPK family. They can be activated in response to various extracellular stimuli and are involved in the regulation of diverse cellular activities, including cell proliferation [44]. The proliferation of cardiac fibroblasts is a crucial factor in the development of cardiac fibrosis. ERK1/2, as part of the MAPK signaling pathway, can respond to various stimulating factors and regulate the structure and function of the heart [45]. To investigate the molecular mechanism of Src-mediated cardiac fibrosis, we conducted experiments to assess the proliferation of cardiac fibroblasts after inhibiting Src. The results showed that inhibiting Src significantly reduced the proliferation of cardiac fibroblasts induced by ISO. While most of the research on Src and cell proliferation has been focused on the field of cancer, where Src overexpression or high activation is often observed, and it acts as a central mediator in many signaling pathways, playing a significant role in promoting tumor cell proliferation [46]. In tumor cells, Src is frequently highly expressed and continuously activated due to the loss of precise negative regulation, contributing to the development of various cancers such as breast cancer and lung cancer [47].

There are certain limitations in our current study. First, cardiac remodeling is a complex process involving the interaction between cardiomyocytes and non-cardiomyocytes. Our study focused on the functional changes of cardiomyocytes and cardiac fibroblasts under ISO exposure alone, without considering the intercellular crosstalk in co-culture systems involving both cell types [48]. It remains unclear whether cardiomyocytes or cardiac fibroblasts play a dominant role in ISO-induced cardiac remodeling. Moreover, cardiac immune cells and surrounding supportive cells also contribute to the cardiac remodeling process, and the importance of intercellular cross-talk and the cellular microenvironment should not be overlooked [49]. Indeed, different cell types exhibit distinct responses when their adrenergic receptors are activated. Our findings demonstrated that Src promotes ISO-induced proliferation and transdifferentiation of cardiac fibroblasts. Conversely, in cardiomyocytes, ISO-induced apoptosis has been observed in previous studies [50]. These observations suggest that the imbalance between increased cardiac fibroblasts and decreased cardiomyocytes is a microscopic manifestation of cardiac remodeling. Interestingly, *in vitro* cell experiments have indicated that Src can mediate the apoptosis process in cardiomyocytes, and the use of the Src inhibitor PP1 can block apoptosis [51]. In the context of sympathetic nerve activation, the intertwined processes of apoptosis, hypertrophy, proliferation, and other cellular fates need to be further elucidated. In addition to the aforementioned limitations, it is worth noting that the phosphorylation of Src and its subcellular distribution play a role in signal transduction within the myocardial nucleus, representing an important process in cellular response to external stimuli [52]. Meanwhile, ERK1/2, as a crucial intracellular mediator, exerts profound effects on cell proliferation, survival, apoptosis and transdifferentiation [53]. The Src/ERK1/2 signaling pathway provides a broad understanding of the mechanisms underlying ISO-induced cardiac remodeling. However, the specificity of Src regulation remains to be fully understood, and specific downstream signaling nodes of Src are yet to be discovered.

In summary, our study has revealed that Src plays a crucial role in mediating ISO-induced cardiac remodeling through the β-AR/Src/ERK signaling pathway. We have demonstrated its involvement in cardiomyocyte hypertrophy, cardiac fibroblast proliferation, and transdifferentiation. By identifying Src as a key molecule influencing cardiac remodeling downstream of β-AR, our findings suggest that Src could serve as a potential drug target for precise modulation of cardiac remodeling.

## Supplementary Material

Supplementary Material S1-S8

## Data Availability

All data are available from the corresponding author upon reasonable request. Original western blots images are available at Supplementary Materials.

## Abbreviations

β-AR: β-adrenergic receptor
AT1R: angiotensin II receptor type 1
EGFR: epidermal growth factor receptor
ERK1/2: extracellular signal-regulated protein kinase
GPCR: G protein-coupled receptor
ISO: isoproterenol
NRCF: neonatal rat cardiac fibroblast
NRCM: neonatal rat cardiomyocyte
PP1: 4-amino-5-(methylphenyl)-7-(t-butyl) pyrazolo-(3,4-d) pyrimidine
ROS: reactive oxygen species
RTK: receptor tyrosine kinase
TNF: tumor necrosis factor
