# Supplementary material for: Src tyrosine kinase promotes cardiac remodeling induced by chronic sympathetic activation
Source: Biosci Rep. 2023 Oct 27;43(10):BSR20231097. doi: 10.1042/BSR20231097 (PMC10611920; doi:10.1042/BSR20231097)
Supplement: Supplementary Material S1-S8 [file BSR-2023-1097_supp.pdf]

Supplementary Materials1. The mortality rate due to isoproterenol in the mice

Supplementary Table1：The mortality rate due to isoproterenol in the mice

The mortality rate

group	CON	ISO	PP1+ISO
sum	10	10	10
number of deaths	0	1	0
mortality rate(%)	0	10	0

Supplementary Table1. Mortality rate of mice during model making.

Supplementary Materials2 .Original western blots of Figure 4A

Figure 4A

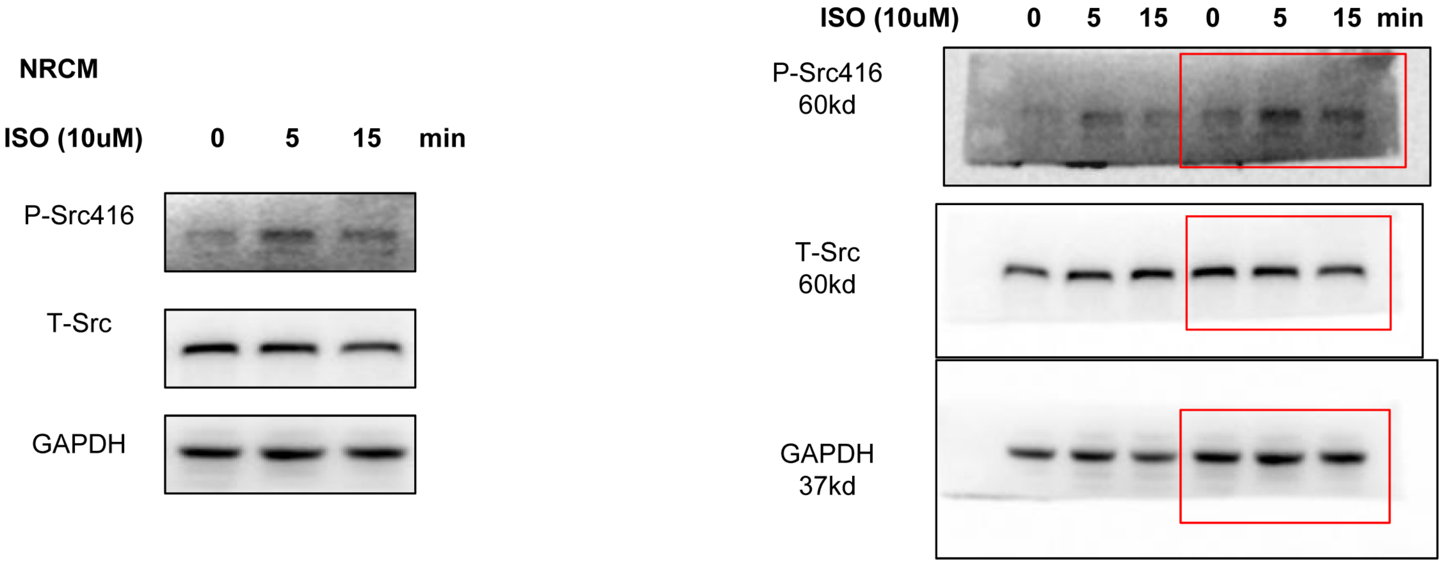

Figure 4A. Src could be activated by isoproterenol. Cardiomyocytes were starved for 12 h, then stimulated with isoproterenol (10 μM) for 5 or 15 min, the phosphorylation of Src was analyzed using Western blotting. Activation of Src was determined by anti-Tyrosine 416 phospho-specific Src antibody. Experiments were performed in four times.

Supplementary Materials3. Original western blots of Figure 5A

Figure 5A

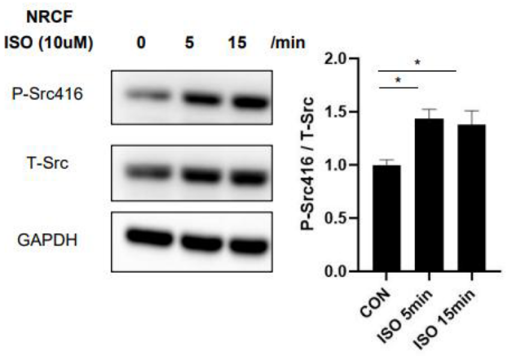

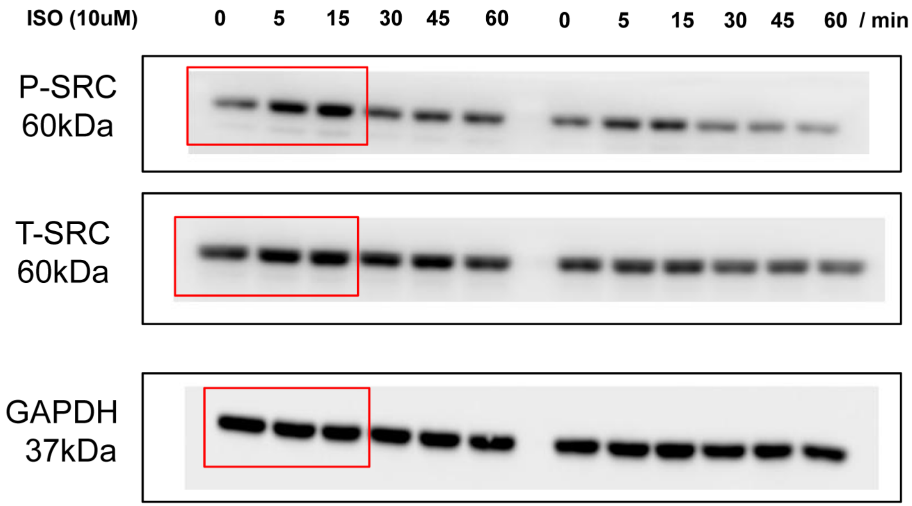

Figure 5A. Src could be activated by isoproterenol. Cardiac fibroblasts were starved for 12 h, then stimulated with isoproterenol (10 μM), the phosphorylation of Src was analyzed using Western blotting. Activation of Src was determined by anti-Tyrosine 416 phospho-specific Src antibody. Experiments were performed in four times.

Supplementary Materials4. Original western blots of Figure 5C

Figure 5C

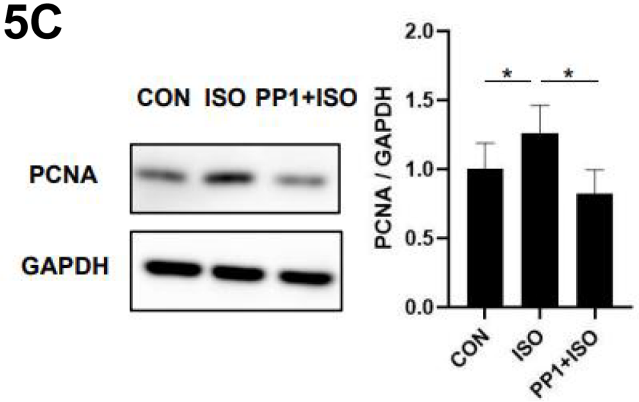

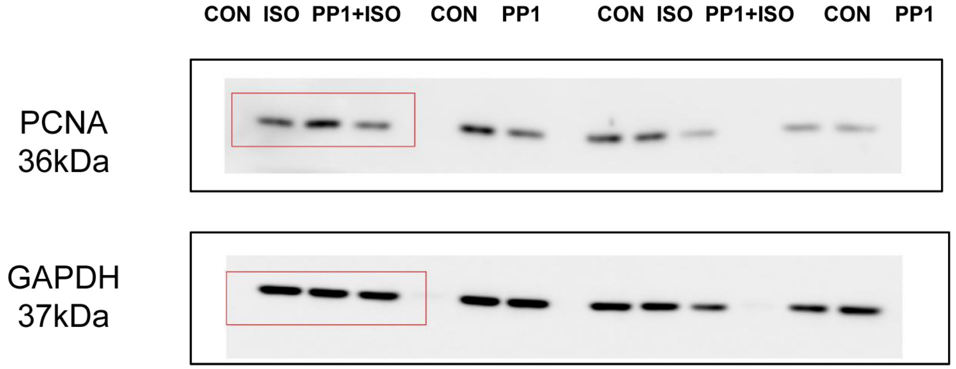

Figure 5C. Inhibition of Src reduces ISO-induced cardiac fibroblasts proliferation indicator PCNA.

Supplementary Materials5. Original western blots of Figure 5D

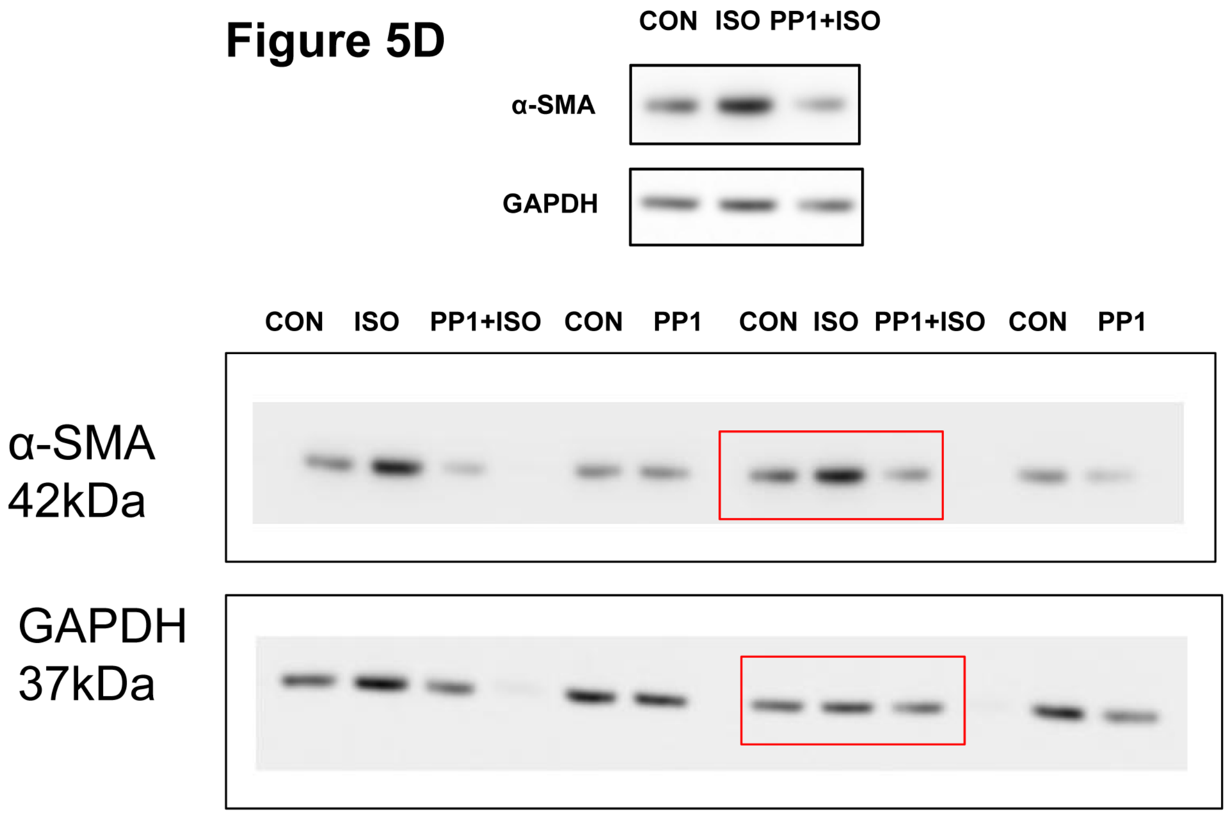

Figure 5D. Inhibition of Src reduces ISO-induced cardiac fibroblasts transdifferentiation indicator α-SMA.

Supplementary Materials6. Original western blots of Figure 6A

Figure 6A

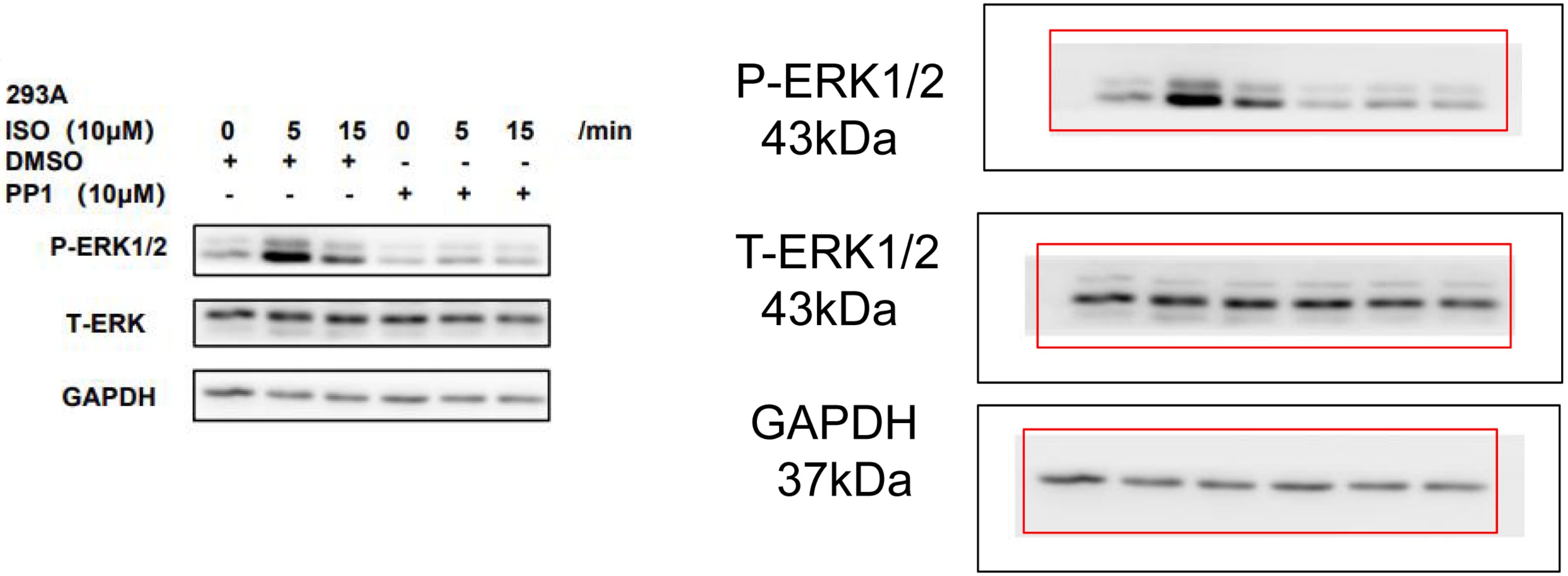

Figure 6A. Inhibition of Src decreases ISO-induced ERK1/2 phosphorylation. HEK-293A was starved for 12 h, treated with or without PP1 (10 μM) for 1 h, then stimulated with isoproterenol (10 μM) for 5 or 15 min.

Supplementary Materials7. Original western blots of Figure 6B with anti-flag

Figure 6B

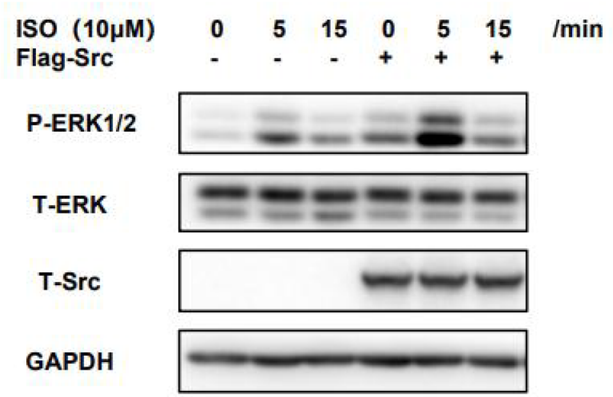

Figure 6B. Overexpression of Src increases ISO-induced ERK1/2 phosphorylation. HEK-293A was transfected with flag-Src plasmid, starved for 12 h, then stimulated with isoproterenol (10 μM) for 5 or 15 min.

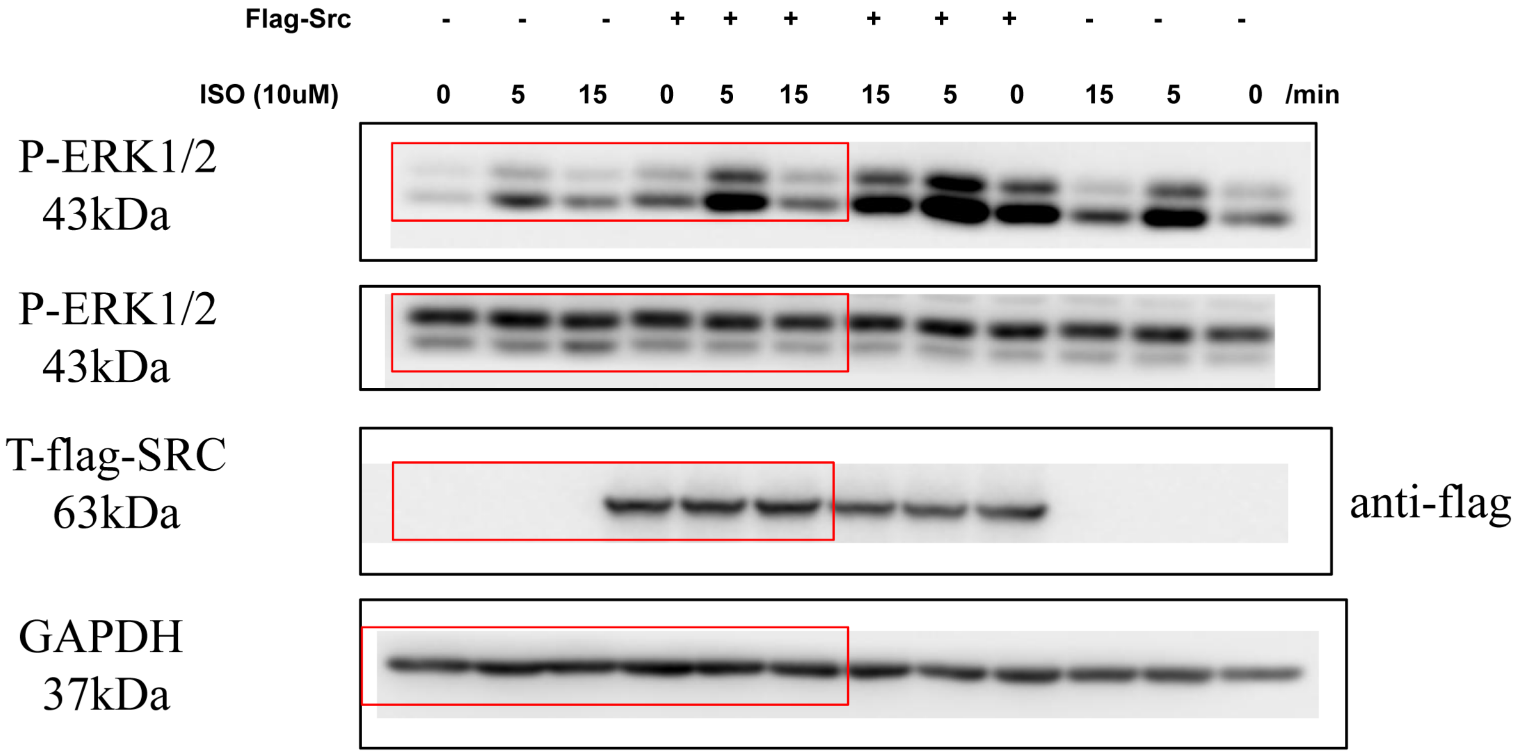

Supplementary Materials7. Original western blots of Figure 6B with anti-T-Src

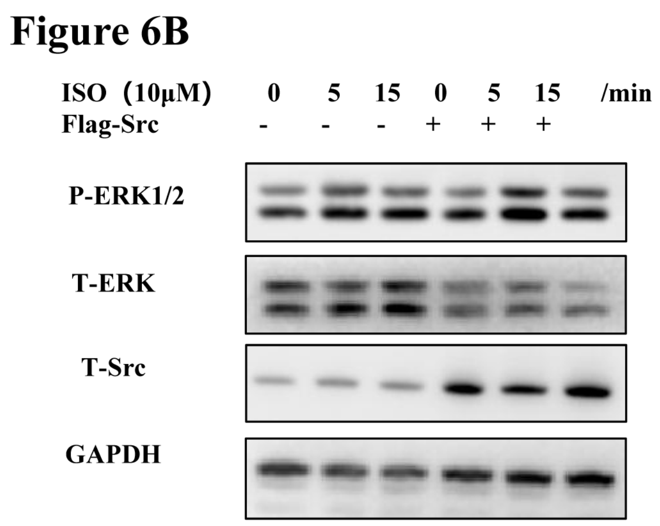

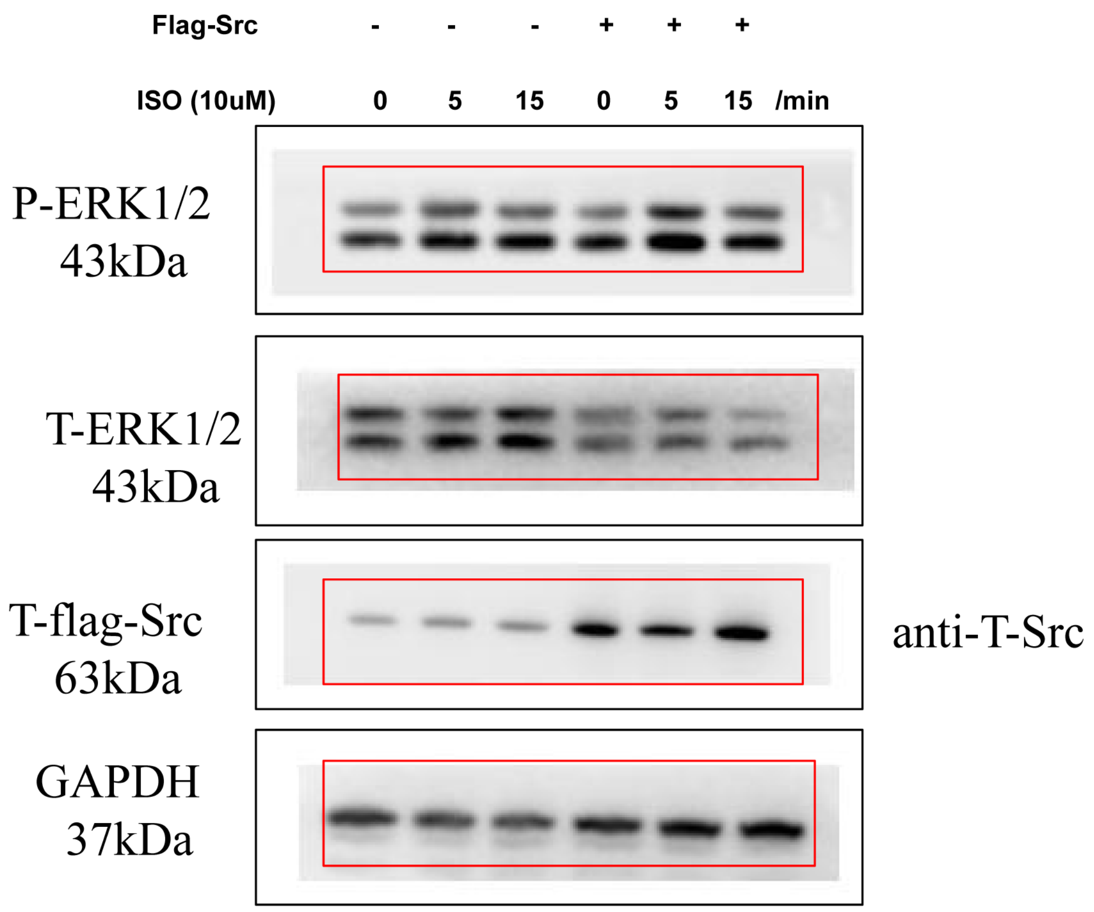

Figure 6B. Overexpression of Src increases ISO-induced ERK1/2 phosphorylation. HEK-293A was transfected with flag-Src plasmid, starved for 12 h, then stimulated with isoproterenol (10 μM) for 5 or 15 min.
